# The long-lasting enigma of polycytidine (polyC) tract

**DOI:** 10.1371/journal.ppat.1009739

**Published:** 2021-08-04

**Authors:** Velia Penza, Stephen J. Russell, Autumn J. Schulze

**Affiliations:** 1 Mayo Clinic Graduate School of Biomedical Sciences, Mayo Clinic, Rochester, Minnesota, United States of America; 2 Department of Molecular Medicine, Mayo Clinic, Rochester, Minnesota, United States of America; 3 Division of Hematology, Mayo Clinic, Rochester, Minnesota, United States of America; NYU Langone Health, UNITED STATES

## Abstract

Long polycytidine (polyC) tracts varying in length from 50 to 400 nucleotides were first described in the 5′-noncoding region (NCR) of genomes of picornaviruses belonging to the *Cardio*- and *Aphthovirus* genera over 50 years ago, but the molecular basis of their function is still unknown. Truncation or complete deletion of the polyC tracts in picornaviruses compromises virulence and pathogenicity but do not affect replicative fitness in vitro, suggesting a role as “viral security” RNA element. The evidence available suggests that the presence of a long polyC tract is required for replication in immune cells, which impacts viral distribution and targeting, and, consequently, pathogenic progression. Viral attenuation achieved by reduction of the polyC tract length has been successfully used for vaccine strategies. Further elucidation of the role of the polyC tract in viral replication cycle and its connection with replication in immune cells has the potential to expand the arsenal of tools in the fight against cancer in oncolytic virotherapy (OV). Here, we review the published data on the biological significance and mechanisms of action of the polyC tract in viral pathogenesis in *Cardio*- and *Aphthoviruses*.

## Features of picornavirus genome

**Fig 1:**
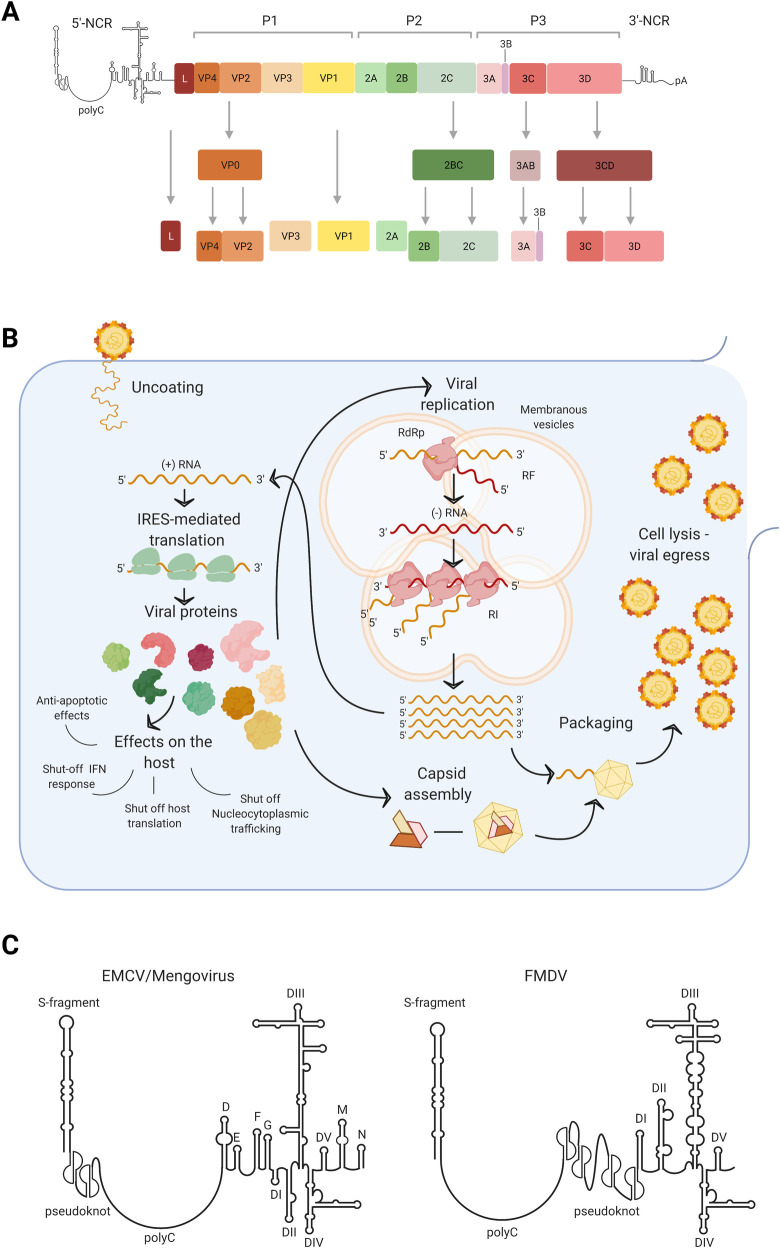
Picornavirus genomic organization, life cycle, and relevant structures of the 5′-NCR. **(A)** Schematic representation of picornavirus genome. Picornavirus positive single-strand RNA genome is composed of 2 NCRs in 5′ and 3′ flanking one open reading frame. The NCRs are highly structured and contain numerous functional RNA elements acting as regulators of the viral life cycle. The coding region encodes for a polyprotein that is co-translationally cleaved into 12–13 final viral proteins and 4 protein precursors by 3C viral proteinase. All picornaviruses share a common organization of the polyprotein into 3 regions: P1, P2, and P3 encoding for 4-3-4 viral proteins, respectively. P1 encodes for 4 structural proteins assembling the capsid, while P2 and P3 contain all nonstructural proteins. Some picornaviruses, including *Cardioviruses* and *Aphthoviruses*, also have an additional Leader (L) protein preceding the P1 region. Created with BioRender.com [1]. **(B)** Picornavirus life cycle. Binding to the cellular receptor triggers uncoating and release of the genomic RNA in the cytoplasm. Here, the IRES recruits the eukaryotic translational machinery to synthesize the polyprotein. The polyprotein is cleaved, leading to the production of single viral proteins. As viral proteins accumulate in the cytoplasm, the host functions are progressively hijacked to favor viral replication. Viral proteins interacting with cellular factors rearrange the membranes of internal organelles to form membranous vesicles, where the viral genome replication complex will be assembled. The RdRp, encoded by the 3D gene, replicates the positive-sense RNA genome into a negative-sense RNA, through a partially double-stranded RNA molecule known as RF. The negative-sense RNA genome is then used as a template for the synthesis by RdRp of many copies of the positive-sense RNA genomes in the RI that can either become the blueprint for more viral protein synthesis, serve as template for replication, or be incorporated in the nascent capsid. The viral capsid protein VP0, VP1, and VP3 auto-assemble into a protomer, assembly of the protomers into higher geometrical structures coordinates the formation of the final icosahedral capsid. After RNA encapsidation, the VP0 precursor self-cleaves into VP2 and VP4, allowing complete maturation of the virion. Viral particles egress occurs upon cell lysis. Created with BioRender.com [1]. **(C)** Schematic representation of the 5′-NCR of *Cardioviruses* EMCV and Mengovirus and *Aphthovirus* FMDV. The 3 species of picornavirus that have been described to carry a polyC tract share a similar organization of the 5′-NCR: The polyC tract is located between a hairpin structure named S-fragment and the type II IRES (domains I to V) and its flanked by pseudoknots. Created with BioRender.com. EMCV, Encephalomyocarditis virus; FMDV, foot-and-mouth disease virus; IFN, interferon; IRES, internal ribosome entry site; NCR, noncoding region; polyC, polycytidine; RdRp, RNA-dependent RNA polymerase; RF, replicative form; RI, replication intermediate.

Picornaviruses comprise a large family of small RNA viruses responsible for a variety of important human and animal diseases. According to the International Committee on Taxonomy of Viruses (ICTV), picornaviruses currently consist of 147 species grouped into 63 genera, and new yet unassigned viruses are continuously identified (http://www.picornaviridae.com/). The best-known genera are *Enterovirus* (poliovirus, rhinovirus, coxsackievirus, and echovirus), *Cardiovirus* (Encephalomyocarditis virus [EMCV] and Theiler’s virus), *Aphthovirus* (foot-and-mouth disease), and *Hepatovirus* (hepatitis A virus). Picornaviruses are non-enveloped viruses of 30 nm in diameter, consisting of an icosahedral capsid containing a tightly packaged non-segmented, single-stranded positive-sense RNA genome that ranges from 7 to 9 kb. The overall organization of the genome is highly conserved across genera: 5′ and 3′ noncoding regions (NCRs) flanking one coding sequence that is translated directly as one polyprotein. The 5′-NCRs range between 400 and 1,500 nucleotides in length, and they are linked at their 5′ terminus to the viral protein VPg, which acts as a primer during viral RNA synthesis. The 3′-NCR is much shorter, usually in the range of 100 to 300 nucleotides, and terminates with an encoded polyadenosine tract [1] (**Fig 1**).

Given the strict volume limitations of the icosahedral capsid, picornaviruses have evolved to pack an extensive repertoire of functions to enter, replicate, and disarm the host cells into a very small genomic space [2]. The coding sequence itself is co- and posttranslationally cleaved by *cis*- and *trans*-acting viral proteinases embedded in the polyprotein itself, resulting in the production not only of the 12 to 13 picornavirus final proteins but also a number of intermediate cleavage products with functions of their own [1]. The number of possible viral factors obtained from a single coding sequence is further increased by the use of alternative partially overlapping open reading frames and ribosomal frameshift to translate cryptic proteins [3,4]. Some of these proteins—such as L, L*, and 2A—are dispensable for viral replication in vitro but are crucial for viral pathogenicity. The concept of “security proteins/virulence factors” has been introduced to describe the role of viral factors that evolved to specifically incapacitate the cellular defensive machinery but are not necessary for viability [5].

Picornavirus genomes also show a remarkable level of functional plasticity at the RNA level. Viral genomes fold into a variety of secondary structures, giving rise to stem-loops, hairpins, clover-like structures, and pseudoknots that can combine or interact with each other to form higher level tertiary structures. The vast majority of the RNA structures identified to date are located in the NCRs at the 5′ and 3′-termini, but important RNA elements are continuously being discovered throughout the viral genomes [6]. These RNA structures, as well as specific RNA sequences and motifs, govern many essential viral processes such as translation, replication, and packaging. For example, a series of stem-loop folds in the 5′-NCR known as the internal ribosome entry sites (IRES) act as a scaffold for the cap-independent assembly of the ribosomal units at the initiation of translation site. Picornavirus IRES elements are classified in type I to IV, the best characterized being type I IRES (*Enteroviruses* and *Rhinoviruses*) and type II IRES (*Cardioviruses* and *Aphthoviruses*). Despite the differences, they all consist of 5 discrete stem-loop structures named modules or domains, which contain binding sites for canonical translation factors and IRES *trans*-acting factors (ITAFs) required for the initiation of viral genome translation [7]. Replication of the viral genome also relies heavily on highly structured RNA domains, which have been described extensively for *Enteroviruses*: Their 5′-NCR harbors a 3-branched cloverleaf structure that guides the assembly of the replication complex that brings together the opposite 5′ and 3′-termini of the genome—a process necessary to shut down translation and initiate negative strand synthesis [8–11]. Another RNA element required for replication is the *cis*-acting replication element or *cre*: This stem-loop structure contains the conserved AAACA motif acting as a template for the addition of uridine residues to viral VPg, which is used by the viral RNA-dependent RNA polymerase as a primer for both negative- and positive-strand synthesis [12]. The *cre* element is frequently found in the coding region of picornaviruses [13,14] with the notable exception of the foot-and-mouth disease virus (FMDV) that harbors it in its 5′-NCR [15]. Finally, although the precise mechanism for encapsidation of picornaviruses has not been completely elucidated, the packaging signal for one group of picornaviruses (*Aichivirus*) has been identified as the stem-loop A of the 5′-NCR [16].

Other RNA motifs and structures are not directly involved in the essential steps of the viral life cycle but play important roles in the pathogenicity of the virus, shaping the symptoms, immunogenicity, persistence, biodistribution, and, eventually, the disease outcome of picornaviruses [17]. Similarly to viral security proteins, these “viral security” RNA elements are dispensable for viability, but crucial for virulence. The most striking examples of RNA alterations modulating pathogenicity come from *Enteroviruses* responsible for causing severe diseases in human. Vaccination efforts against poliovirus using the live-attenuated Sabin 3 strain led to the discovery that a single point mutation at position 472 on domain V of the type I IRES is a major determinant of poliovirus neurovirulence and can quickly revert to wild type upon replication in the human gut, as shown by cases of vaccine-associated poliomyelitis [18]. Another species under the *Enterovirus* genus, Coxsackievirus group B (CVB) is known to cause acute myocarditis in human, which can be fatal. This disease can sometimes lead to a chronic inflammation of the cardiac tissue associated with the presence of CVB2 and CVB3 genomes carrying small 5′-terminal RNA deletions of approximately 10 to 50 nucleotides [19]. These deletions map to the stem-loop I of the cloverleaf and have been shown to decrease—but not completely abolish—the affinity of the structure for cellular and viral replication factors, PCBP2 and 3CD^pro^ [20,21]. The molecular mechanism leading to this switch from acute to chronic CVB infection of human cardiac tissue is not completely understood, but these data point to alterations of 5′-NCR structures being responsible for the covert survival of CVB on low-replication mode in the heart long after the acute infection has subsided. Another way RNA structures can affect the pathogenicity is by mediating viral immune evasion. The 350 to 380 nucleotides at the far end of FMDV 5′-terminus are known as the S-fragment, and they fold into a long hairpin structure [22]. Full-length S-fragment carries a molecular signature that prevents induction of the innate immune response, while deletions of the exposed loop enhance production of interferon beta (IFNβ), interferon-stimulated genes (ISGs), and inflammatory cytokines in vitro and attenuate the virus in vivo, immunizing mice against further challenges with wild-type FMDV [23,24].

Long stretches of 50 to 400 cytidine residues in a row, sporadically interrupted by species-specific uridine discontinuities, have been identified in selected species of picornaviruses, all belonging to the *Cardioviruses* and *Aphthoviruses* genera. More specifically, the best-known bearers of this peculiar RNA element are the FMDV in the *Aphthovirus* genus, EMCV, and its sub-strain Mengovirus for the *Cardiovirus* genus. These polycytidine (polyC) tracts have drawn attention since their discovery because the evolutionary burden of maintaining such a long repetitive noncoding sequence intuitively implies a vital function. Extensive studies have shown that the polyC is most likely another example of such “viral security” RNA elements: Deletion or truncation of the tract does not affect the viability of the viruses but greatly diminishes their virulence. The role as pathogenicity determinant, the genetic stability, and the impact on viral viability of the polyC tract differ greatly from virus to virus, which makes it even more challenging to draw conclusions on the exact function and molecular mechanism of attenuation.

## Polycytidine tract in the 5′-NCR of picornavirus

The presence of a discrete polyC tract within the genomic RNA of picornaviruses was first reported in EMCV as a tract of about 100 nucleotides in length [25].This observation was later confirmed and expanded to include picornaviruses of the *Cardiovirus* and *Aphthovirus* genera, namely several strains of FMDV and a sub-strain of EMCV called Mengovirus [26]. More recently, 2 other members of the *Aphthovirus* genus, equine rhinitis A virus (ERAV) and bovine rhinitis B virus (BRV-2) were reported to have polyC tracts [27,28]. Equine rhinitis B virus (ERBV), recently reclassified from *Aphthovirus* to *Erbovirus* genus, also carries a polyC tract [29].

These long stretches of polyC repeats all share the same location within the genome of the picornavirus in which they have been described, within the first 500 nucleotides of the 5′-NCR. More specifically, the polyC is localized after the first 150 nucleotides for EMCV and Mengovirus [30,31], after the first 400 nucleotides for FMDV [32,33], and after approximately 300 nucleotides for ERAV and ERBV [27].

Variation in the length of this tract among isolates and among subclones of lab strains is considerably high. FMDV polyC tract ranges from 150 to 250 nucleotides in natural isolates and shows even more variability in lab and vaccine strains [26,34–37]. *Cardioviruses* have shorter polyC tracts, with EMCV varying from 70 to 250 nucleotides and Mengovirus isolates ranging from 50 to 100 nucleotides [25,26,35,38]. Interestingly, uridine discontinuities have been described for *Cardioviruses*: C_n_UC_10_ for Mengovirus and C_n_UCUC_3_UC_10_ for EMCV (**Table 1**). Accurate sequencing data for the much longer polyC tracts in *Aphthoviruses* strains is not available to date; therefore, the length of the polyC tract in natural strains of these viruses has only been estimated through RNAse digestions and is reported as an uninterrupted polypyrimidine segment. Consequently, it is not known whether these discontinuities are a peculiarity of *Cardiovirus* genus (**Table 1**). Similarly, the complete sequence of the polyC tract and the entire sequencing upstream of it have not been determined in ERAV, BRV-2, and ERBV; therefore, the total length of the polyC is unknown, but runs of at least 13 to 17 cytidine residues have been reported for all of them [27–29].

While other “viral security elements” in picornavirus genomes consist of highly structured RNA, polyC tracts in FMDV and EMCV are largely unstructured: Biochemical analysis showed that the polyC tract is mostly looped out and exposed, therefore not forming any secondary structure or pairing with surrounding regions [39,40].

**Table 1:** Summary of polyC tract variants reported for *Cardioviruses* (EMCV and Mengovirus) and *Aphthoviruses* (FMDV) strains.

EMCV and Mengovirus									
Strain name	GenBank access number	Reference	Country of origin	Species of origin	Flanking 5′ (23 nt)	PolyC sequence	Flanking 3′ (20 nt)	Total polyC length	Source of sequencing
EMCV-R	NC_001479.1	[66]	United States of America	Chimpanzee 5 yo fatal myocarditis	TGCCACCCCAAAATAACAACAGA	C_115_UCUC_3_UC_10_	TAACGTTACTGGCCGAAGCC	132	vRNA
EMCV-D	M22458	[60]	-	(pig)	TGCCACCCCAACATAACAACAGA	C_130_	AACGTTACTGGCCGAAGCC	130	vRNA
EMCV-B	M22457	[60]	-	(pig)	TGCCACCCCAACATAACAACAGA	C_127_	AACGTTACTGGCCGAAGCC	127	vRNA
EMCV-PV2	X87335	[62]	-	(pig)	TGCCACCCCAACCAACAAAACAAAAA	C_118_	AACGTTACTGGCCGAAGCC	118	vRNA
EMCV-PV21	X74312.1	[59]	-	(pig)	TGCCACCCCAAAACAACAACAGA	C_141_UCUC_3_UC_10_	TAACGTTACTGGCCGAAGCC	158	vRNA
EMCV-BEL-2887A	AF356822	[44]	Belgium	Aborted swine fetus	TGCCACCCCAAAACAACAACAAA	C_10_UCUC_3_UC_10_	TAACGTTACTGGCCGAAGCC	27	vRNA
EMCV-30/87	AY296731	[57]	USA	Aborted swine fetus	TGCCACCCCAAACAACAACAACAAAACAAACT	C_5_UC_8_	TTACTATACTGGCCGAAGCC	14	cDNA
EMCV-BJC3	DQ464062.1	[53]	China	Aborted swine fetus	TGCCACCCCAAAACAACAACAAA	C_9_UCUC_3_UC_10_	TAACGTTACTGGCCGAAGCC	26	cDNA
EMCV-HB1	DQ464063.1	[53]	China	Heart of piglet with myocarditis	TGCCACCCCAAAACAACAACAA	C_7_UCUC_3_UC_10_	TAACGTTACTGGCCGAAGCC	24	cDNA
EMCV-K13	EU780148	[54]	South Korea	Mother of aborted swine fetus	TGCCACCCCAAAACAACAACAAA	C_13_	TAACGTTACTGGCCGAAGCC	13	cDNA
EMCV-K11	EU780149	[54]	South Korea	Aborted swine fetus	TGCCACCCCAAAACAACAACAAA	C_7_UCUC_3_UC_10_	TAACGTTACTGGCCGAAGCC	24	cDNA
EMCV-CBNU	DQ517424	[55]	South Korea	Aborted swine fetus	TGCCACCCCAAAACAACAACAAA	C_10_	TAACGTTACTGGCCGAAGCC	10	cDNA
EMCV-HNXX13	MH191297	[56]	China	Aardvark	TGCCACCCCAAAATAACAACAAA	C_7_UCUC_3_UC_10_	TAACGTTACTGGCCGACGCC	14	cDNA
Mengovirus strain M	L22089.1	[38]	Uganda	Paralyzed rhesus monkey	TGCCAACCCAAAACCACATAA	C_50_UC_10_	TCACATTACTGGCCGAAGCC	61	vRNA
Mengovirus strain 3761IMP	KX231802.1	[52]	Russia	Hamadryas baboons (*Papio hamadryas*)	TGCCACCCCAAAGTACACAA	C_8_	GTACATTACTGGCCGAAGCC	8	cDNA
FMDV									
**Strain name**	**GenBank access number**	**Reference**	**Country of origin**	**Species of origin**	Flanking 5′ (20 nt) (*)	**PolyC sequence**	Flanking 3′ (20 nt) (*)	**Total polyC length**	**Source of sequencing**
FMDV C3 Resende, clone 12	AY593807.1	[64]	/	/	CGCCCGAAACCCGCCTTTCA	C_230_	TAAGTTTTACCGTCGTTCCC	230	vRNA
FMDV C3 Resende, clone 3B	AY593807.1	[64]	/	/	CGCCCGAAACCCGCCTTTCA	C_145_	TAAGTTTTACCGTCGTTCCC	145	vRNA
FMDV A-61	MN227144.1	[26]	/	/	ACCCGGCGCCCGCCTTTCAT	C_150_	TAAGTTTTACCGTCGTTCCC	150	vRNA
FMDV O-V1	NC_039210.1	[26]	/	/	CACCCGAAGCCCGCCTTTCA	C_150_	TAAGTTTTACCGTCGTTTCC	150	vRNA
FMDV C-GC	Not available	[26]	/	/	/	C_200_	/	200	vRNA
FMDV C-997	Not available	[26]	/	/	/	C_150_	/	150	vRNA
FMDV SAT1, passage 7	MN275121.1	[36]	/	/	ACCTGAATGCCTGCCTTTCA	C_170_	GAACGATGCCGTCTTTCCCG	170	vRNA
FMDV SAT1, passage 82	MN275121.1	[36]	/	/	ACCTGAATGCCTGCCTTTCA	C_100_	GAACGATGCCGTCTTTCCCG	100	vRNA
FMDV Lausanne 1965	Not available	[34]	Switzerland	/	*/*	C_120_	*/*	120	vRNA
FMDV UK 18/81	Not available	[34]	United Kingdom	/	*/*	C_120_	*/*	120	vRNA
FMDV UK 1848	Not available	[34]	UK	/	*/*	C_100_	*/*	100	vRNA
FMDV India 53/79	Not available	[34]	India	/	*/*	C_200_	*/*	200	vRNA
FMDV Thailand 1/80	Not available	[34]	Thailand	/	*/*	C_100_	*/*	100	vRNA
FMDV C-S8c1, passage 100	Not available	[37]	/	/	*/*	C_420_	*/*	420	vRNA

(*) sequence of specific strain not available, serotype sequence used.

cDNA, complementary DNA; EMCV, Encephalomyocarditis virus; FMDV, foot-and-mouth disease virus; nt, nucleotides; polyC, polycytidine; vRNA, viral RNA.

The absence of this tract in other genera of the picornavirus family such as *Rhinovirus* and *Enterovirus* suggests that the polyC fragment is a distinctive feature of *Cardioviruses* and *Aphthoviruses*. Interestingly, the general landscape of RNA structures and motives making up the 5′-NCRs of these viruses is extremely similar and sets them aside from other picornaviruses. In both genera, the very 5′-end of the NCR folds into a long hairpin structure known as the S-fragment, while the region immediately preceding the polyprotein is organized into IRES type II. The space in between these 2 RNA elements contains the polyC tract, flanked on either side by a series of pseudoknots (**Fig 1**). The fact that this specific combination of structures is conserved in picornaviruses carrying the polyC tract might suggest that this RNA element needs to act in concert with other RNA elements to perform its function.

## Effects of polyC tract alterations in picornaviruses

### Viability

First attempts at cloning full-length genomes for EMCV and Mengovirus led to the serendipitous discovery that infectious clones carrying substantial or even total deletions of the polyC tract generate viable viruses that replicate with the same kinetics and titers of the wild type in vitro, although forming slightly smaller plaques [38,41–44]. The presence of a polyC tract is not an absolute requirement for FMDV viability either. Infectious clones with a minimal number of 2 cytidine residues are still able to rescue the virus in baby hamster kidney (BHK) cells [45]. This observation lead to the initial conclusion that the polyC tract is dispensable for the reproductive cycle of the virus, which was later disputed by studies on polyC effect on pathogenicity.

### Genetic stability

PolyC tracts lengths vary in a virus specific manner: around 50 nucleotides for Mengovirus, 100 to 150 for EMCV, and 100 to 400 for FMDV (**Table 1**). This variability is expected in RNA viruses, which notoriously have a high mutational and recombination rate [46], and it is in agreement with the idea that the polyC does not require an exact number of cytidine residues to perform its function but rather conserves an overall length, which varies from virus to virus. The fact that natural isolates with severely truncated polyC tracts have never been reported speaks to the importance and stability of this genetic feature in *Cardio*- and *Aphthoviruses* in the wild and suggests the presence of a mechanism to preserve the length of the polyC in an optimal range.

Studies on FMDV support these assumptions on polyC genetic stability. Recombinant FMDV with a polyC of only 2 cytidines (polyC_2_) is viable in BHK cells and has proven to be virulent in a nonhost species, but it is not genetically stable as it evolved deletion mutants directly downstream of the 2 cytidines [45]. Infectious clones carrying slightly longer polyC tracts of 6 cytidine residues consistently rescued viruses where the polyC tract is immediately amplified to 60 to 150 nucleotides [45,47]. This interesting phenomenon seems to imply that (i) FMDV relies on a polyC-tract amplification mechanism—such as polymerase slippage or recombination, which requires a minimum number of consecutive cytidine residues greater than 2; and (ii) FMDV with longer polyC tracts have a selective advantage and rapidly and efficiently outcompete shorter variants in the span of few replication cycles. Infection of bovine and swine cell lines, natural FMDV hosts, by a mixed population of FMDV strains carrying different length of the polyC tracts similarly led to the enrichment of viruses with the longest polyC tract [48]. In steers, the acute phase of FMDV infection is defined by the virus predominantly replicating in vesicular lesions at peripheral sites in the oral mucosa and coronary bands of the feet [49]. When steers were infected with a mixed population of FMDV variants differing only by the length of their polyC tracts, longer-tract FMDVs rapidly established themselves as the prevalent viral species isolated from these vesicular lesions [48]. In roughly 50% of cases, FMDV infection can progress into a chronic subclinical phase where replication is restricted only to the lymphoid-associated epithelium of nasopharyngeal mucosa [49]. Infection and persistence in the nasopharyngeal mucosa were shown to be associated with gradual and progressive increases in the length of the polyC of the inoculated virus that started as early as 7 days postinfection [48]. These observations indicate that elongation of the polyC tract is not just occurring in vitro but is likely a functionally relevant tissue-specific adaptation associated with FMDV persistence in cloven-hoofed animals.

PolyC tract truncations and deletions in *Cardioviruses* are remarkably more stable than in FMDV. Both recombinant EMCV and Mengovirus with shorter polyC tracts can be rescued faithfully from infectious clones [38,42]. Truncated MC_24_ (C_13_UC_10_) and the deleted MC_0_ (no polyC) variants of Mengovirus, similarly to their EMCV-R counterparts C_4_, C_9_ and C_20_, do not show evidence of polyC length reversion even after extensive passaging in vitro [38,42]. It should be noted although that their genetic stability has exclusively been tested in cell lines where the polyC tract length does not influence the replication kinetics at all, and, therefore, no selective pressure is present on short-tract viruses. In vivo passaging of Mengovirus variant MC_24_ (C_13_UC_10_) in mice brains similarly failed to restore virulence, as none of the passages induced symptoms of Mengovirus infections in mice [50]. However, since the length and sequence of the polyC was not assessed directly for each passage, it is difficult to speculate whether the polyC tract length is stably maintained or an amplification mechanism is acting on the polyC tract to generate a heterogeneous population that has not reached the pathogenic threshold yet [50]. Sequencing of MC_24_ administered as oncolytic therapy to tumor-bearing mice showed that short (C_13_UC_10_) polyC tracts can remain stable up to 4 days postinfection [51]. However, the replication of oncolytic MC_24_ had also been restricted by the addition of microRNA (miRNA)-targeting elements, recognized by cognate miRNA overexpressed in cardiac and neuronal tissues to further limit toxicity. Such restriction of viral replication might influence the virus’ ability to amplify the polyC tract and escape [51]. The use of short-tract Mengovirus and miRNA targeting in oncolytic virotherapy (OV) is further discussed in the Applications section.

### Pathogenicity

Attempts at establishing a connection between pathogenicity of natural isolates and length of the polyC tracts are intrinsically hindered by the difficulty of correctly assessing the length of the polyC. Reports of pathogenic Mengovirus, EMCV, and FMDV natural isolates carrying short polyC tracts are biased by the methodology used to sequence the tract: Reverse transcriptase and other template-dependent DNA polymerases have difficulty reproducing repetitive sequences with fidelity and inevitably shorten the tract, resulting in cDNA fragments used for sequencing that do not represent faithfully the length of the polyC tract present in the natural isolates. This is reflected by the fact that every time EMCV polyC tract sequencing has been attempted using cDNA intermediates, it resulted in a short polyC tract ranging from 8 to less than 40 nucleotides, while direct assessment of polyC tract length as it appears in the viral RNA through RNase digestion and radiolabeling always results in long tracts ranging from 110 to 160 nucleotides (**Table 1**) [52–56]. Consequently, the existence of natural short-tract isolates is questionable, and statements about the effects of polyC tract length on the pathogenicity of these viruses based on sequencing through cDNA intermediates are not reliable.

Given this technical difficulty, it is not surprising that a clear relationship between polyC tract length and pathogenicity has only been established for Mengovirus, which carries the shortest—and, therefore, more easily manipulated—polyC tract.

Comparisons between natural long-tract isolates and recombinant short-tract EMCVs show contradicting results, making it hard to establish a linear relationship between polyC length and pathogenicity. A thorough analysis of the role of the polyC tract length in EMCV-R strain (C_115_UCUC_3_UC_10_) using constructs carrying polyC tracts of 4, 9, or 20 nucleotides shows that even important truncations of the polyC tract only cause mild attenuation of EMCV in mice [42,57]. However, comparisons of polyC length variants using other EMCV strains as backbone have shown opposite results. An infectious clone obtained from the pathogenic strain EMCV-2887A carrying a short polyC tract of 27 nucleotides total (C_10_UCUC_3_UC_10_) was 100 times more attenuated in mice than its wild type counterpart [44]. Similarly, a recombinant virus differing from its parental EMCV-HB10 strain only for the length of the polyC tract (C_9_) showed a 200-fold attenuation [58]. Infectious clones derived from the extremely pathogenic EMCV-PV21 strain and designed to carry a long polyC tract of approximately 150 nucleotides—close to the natural isolate 158 nucleotide–long polyC tract C_141_UCUC_3_UC_10_—showed the same levels of pathogenicity, virulence, and biodistribution of their parent strain in NMRI mice [59].

Even though the impact of the polyC tract length on EMCV virulence might not be clear when the outcome is measured purely by survival, alterations in the length and sequence of the polyC tract can induce target tissue-specific variations that ultimately affect the pathological progression of EMCV in mice. The diabetogenic strains EMCV-D and EMCV-D/PV2 have been reported to carry long polyC tracts of 130/144 nucleotides and 118 nucleotides, respectively [60–62]. Attempts at producing infectious clones for these strains using reverse transcription on the polyC tract resulted in recombinant viruses carrying much shorter polyC tracts of only 34 cytidines and 20 cytidines that no longer cause diabetes in susceptible mouse models [43,63]. EMCV-D/PV2 and EMCV-D/PV2 polyC_20_ titers reach comparable levels in the pancreas within the first 5 days of infection; however, the parental strain EMCV-D/PV2 causes severe insulitis and necrosis, while EMCV-D/PV2 polyC_20_ induces only mild histomorphological changes in Langerhans islets [63]. Differences in infection and pathological lesions also extend to EMCV classical target tissues, the heart, and the brain. Both viruses replicate to high titers in the heart and cause extensive myocarditis with necrosis and immune cell infiltrations, but only the parental strain EMCV-D/PV2 reaches high replication titers in the brain [63]. Interestingly, substitutions of the polyC tract with a random filler sequence or a polyuridine (polyU) tract of equal length in EMCV-D/PV2 similarly reduces replication in the brain and pathological lesions in the pancreas, without affecting the myocardial infection [63].

The length of the polyC tract in FMDV does not unequivocally correlate with pathogenicity in the host either. FMDV strains differing uniquely for the length of their polyC tract are equally pathogenic in cattle [64]. Even though viral attenuation in cattle can sometimes associate with a reduction of the polyC tract length, as in the case of the vaccine strain FMDV SAT1-82 [36], this is not a consistent trend, as shown by the highly attenuated FMDV-R100 strain, which carries a polyC tract of 420 nucleotides—the longest ever reported for FMDV [37]. However, it should be noted that attenuated FMDV-R100 carries several other mutations in other key RNA structures like the IRES, which might be directly responsible for the attenuation [37]. FMDV rescued from infectious clones carrying a polyC tract of only 2 cytidines, although not genetically stable, is still virulent in mice [45]. The pathogenicity of this FMDV polyC_2_ has never been tested in the natural host, where the presence of the polyC tract might be necessary to establish a successful infection [45].

The polyC tract is unequivocally a major determinant of viral pathogenicity for Mengovirus. Mengovirus is neurotropic and induces a rapid and lethal meningoencephalomyelitis after intraperitoneal or intracerebral injection, with onset of severe neurological symptoms within the first week postinoculation and death by day 10 in mice [65]. The M isolate of Mengovirus polyC tract is 55 nucleotides long with the sequence C_44_UC_10_ [38]. Mengovirus variants of the M isolate carrying a total deletion of the polyC (MC_0_) or a truncated polyC C_13_UC_10_ (MC_24_) are dramatically attenuated in mice in terms of lethal outcome, disease severity, and neuropathogenic properties [41,66–68]. The attenuation in mice varies depending on several factors like mouse strain, age, and route of administration of the virus, but the most interesting observation is that it also progresses linearly with the extent of the polyC tract deletion, so that the shorter the polyC tract, the more attenuated the virus [41,66–68]. The steepest reduction in virulence measured as LD50 (dose causing 50% mortality within 14 days postinoculation) occurs between 40 and 24 cytidines, which suggests that the minimal length required for function might be found at this threshold [41,67]. The reduced virulence of short-tract and polyC-deleted Mengovirus variants is not restricted to mice but extends to other animals known to be naturally infected by both Mengovirus and EMCV, like baboons, macaques, domestic pigs, and a wide variety of non-murine mammals and zoo animals known to be susceptible to infections [69,70].

Neuropathological properties of Mengovirus inversely correlate with the polyC tract length. Direct intracranial administration has shown that wild-type Mengovirus replicates extensively in the brain until death of the animal, causing acute encephalitis as early as 1 day postinfection, with macrophage, lymphocyte and leukocyte infiltration, and necrosis of neuronal cells [50,66]. Short-tract Mengovirus MC_24_ has drastically reduced viral replication in the brain and induces only mild neuronal lesions, while the polyC-deleted Mengovirus MC_0_ reaches even lower titers in the brain and causes no detectable histopathological changes [50,66].

Effects on viral replication in the brain are paralleled by similar effects on viremia. Infection with wild-type Mengovirus causes high and sustained viremia in mice until death [71]. Intraperitoneal or intramuscular inoculation of mice, baboons, and macaques with truncated or deleted polyC tract only occasionally results in a mild and transient viremia that resolves in a few days [50,69].

In spite of poor replication rates, short-tract Mengovirus with total or partial deletions of the polyC tract are excellent immunogens: Sublethal doses of the virus lead to development of neutralizing antibodies in mice, baboons, macaques, pigs, and a variety of other mammals [50,66,69,70]. The onset, the timing, and the magnitude of the final antibody titer depend to some extent on the initial dose, but once the effective threshold is reached, all animals seroconvert within 2 to 3 weeks from inoculation, whether the virus was delivered by intracranial, intraperitoneal, intramuscular, or subcutaneal administration [50,69]. Most importantly, animals exhibiting any level of neutralizing antibodies are then protected long-term against otherwise lethal reinfections, not only with wild-type Mengovirus but also with EMCV, which has 95% of amino acid sequence identity with Mengovirus [50,69].

In addition to invoking a potent humoral immune response, short-tract Mengovirus infection elicits a cell-mediated immune response. Passive transfer of serum from immunized to naive mice is protective against challenge with EMCV but only if the neutralizing antibody titer is above a certain threshold [72]. On the other hand, splenocytes enriched in CD4+ T cells from immunized mice are capable of adoptively transferring immune protection against lethal EMCV challenge even in the absence of prophylactic levels of serum neutralizing antibodies [72]. Similarly, major histocompatibility complex (MHC) class II–deficient RHAβ^−/−^ mice, which have negligible CD4+ T cells and are incapable of producing neutralizing antibodies, even though more susceptible to MC_24_ infection can still survive and mount a protective CD8+ T cell–mediated immune response against subsequent rechallenges with EMCV and Mengovirus, even though this protection is short lived and starts to wane after 90 days postinoculation [73].

Variations in the length of the polyC tract also seem to affect the innate immune response to Mengovirus. Mengovirus, as all *Cardioviruses*, is extremely sensitive to the antiviral response generated by type I IFN pathway activation [74]. Infection with short-tract Mengovirus alters the IFNα/IFNβ release profile both in vivo and in vitro, resulting in a reduction of these circulating cytokines that goes in lockstep with the reduction in viremia levels [41,68]. The screening of a panel of murine cell lines has shown that immune cells of hematopoietic origin—dendritic cells, B and T lymphocytes, and, especially, macrophages—are permissive for short-tract Mengoviruses but significantly slow down and reduce their replication and cytotoxicity [41]. Moreover, immunosuppression in mice induced by repeated administration of cyclophosphamide unexpectedly reduces replication of wild-type Mengovirus in the brain while having little to no effect on short-tract Mengovirus replication [68].

Together, these preliminary findings suggest the interesting hypothesis that a long polyC tract might be a cell type–specific requirement to establish a successful infection of host immune cells, either to directly squash the antiviral immune response or to hijack immune cells to facilitate and spread the infection. What makes this idea appealing is that there is considerable evidence supporting the role of immune cells, especially monocytes and macrophages, as vessels for viral replication, persistence, and dissemination in viral pathogenesis [75]. In case of EMCV, the virus is able to establish chronic infection in mice by directly infecting tissue-resident macrophages of the lungs and thymus [76,77]. Inflammatory mediators produced by infected macrophages deeply influence pathogenesis, causing disruption of β-cells in diabetogenic EMCV [78]. Depletion of macrophages, CD4+, and CD8+ T lymphocytes limits the onset of spinal cord lesions and consequent early and late hind limb paralysis during EMCV infection [79,80]. In contrast, the relationship between immune cell infection and viral pathogenesis and persistence is not as straightforward in the case of FMDV. Primary infection occurs at the level of epithelial cells of the nasopharyngeal mucosa (in cattle) or oropharyngeal tonsil (in pigs), from which the virus can gain access to antigen presenting cells, T lymphocytes, and natural killer (NK) cells [49]. Deep lymphopenia and functional alteration of lymphocytes, dendritic cells, and monocytes/macrophage as well have been reported, but the ability to infect immune cells and cause functional alteration vary in a host- and viral strain–dependent manner [81]. As much as the explanation of polyC tract as a molecular gateway for immune cell access is intriguing, not enough evidence is available at the moment to support it.

## Molecular mechanism of the polyC tract: Unwinding a 50-year old enigma

Collectively, these data suggest that the polyC-mediated impact on virulence is heavily dependent on a host component capable of detecting this small genomic difference early during infection. The unaltered replication kinetics in vitro, modest replication in the brain and low viremia suggest that short-tract viruses are viable and capable of replicating similar to their wild type counterparts. However, at each infectious cycle, they lose more ground against the cumulative effects of innate and adaptive immune responses until the host immune system eventually wins the arms race and clears the infection. Full-length polyC tracts somehow enhance the viral pathogenic effect and are capable of overpowering the host immune system.

**Fig 2:**
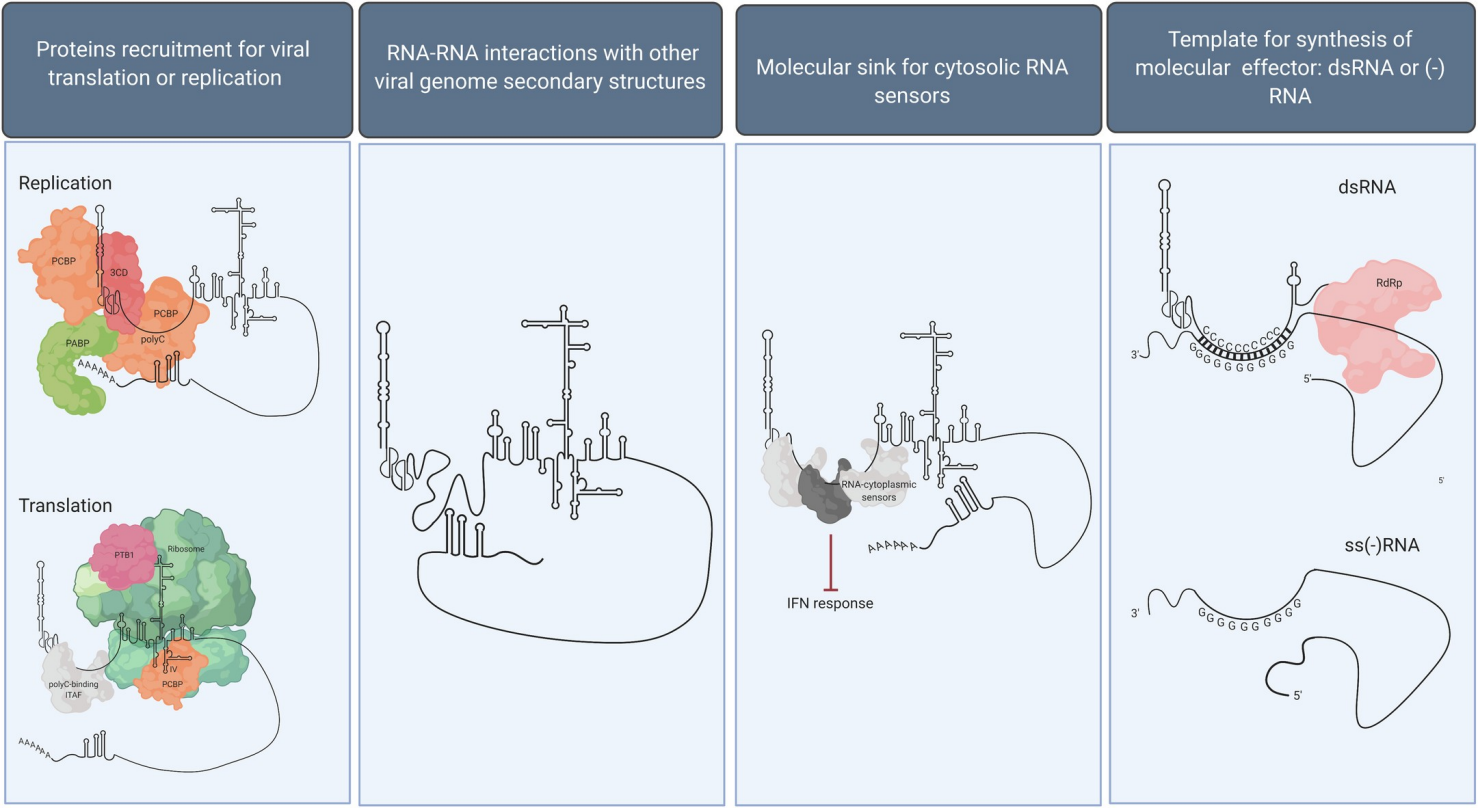
Possible molecular mechanisms mediating the polyC function. Detailed discussion of each potential molecular mechanism is included in the text. Created with BioRender.com. IFN, interferon; ITAF, IRES trans-acting factor; PABP, polyA-binding protein; PCBP, poly(rC)-binding protein; polyC, polycytidine; PTB, polypyrimidine tract–binding protein.

These findings highlight an important biological difference between Mengovirus on one side and the EMCV and FMDV on the other, which is especially surprising when we consider that Mengovirus is a sub-strain of EMCV. Such a difference might be explained by the fact that Mengovirus has a much shorter polyC tract, and, therefore, the biological effects of length variations are immediately apparent, while EMCV and FMDV long polyC tracts are capable of buffering much more loss in sequence. Another possible explanation is that EMCV and FMDV might have evolved redundant systems to perform the same function that in Mengovirus is associated with the polyC alone, and, therefore, are more readily capable of overcoming variations in length. An interesting observation comes from studies on chimeric Mengo-EMC viruses: Replacing the first 411 nucleotides of the 5′-NCR containing the polyC tract of EMCV with the equivalent sequence of the attenuated Mengovirus, MC_24_, results in an extremely attenuated virus, which behaves in vivo just like its Mengovirus counterpart, whereas simply reducing the number of cytidine residues to C_20_ in EMCV has very little effect [42]. This suggests that the genomic context of the polyC tract is just as important for attenuation as the polyC tract itself and that whatever mechanism mediates attenuation probably involves motifs and/or structures from the surrounding sequences too. It is worth noting that the polyC tract has been described in viruses that share a very similar organization of the 5′-NCR, with a long hairpin stem-loop and 2 to 4 pseudoknots surrounding the polyC tract (**Fig 1**). However, the evolutionary burden of maintaining such a long repetitive polyC tract in the face of restrictive genomic space constrains implies the existence of a fitness payoff. The function and the molecular mechanism at the basis of the polyC tract still elude us, but some speculations can be made (**Fig 2**).

The first scenario sees the polyC tract as a binding site for cellular or viral factors involved in replication, translation, or packaging. Constructs comprised of type II IRESs derived from polyC-containing picornaviruses are routinely used in monocistronic and bicistronic vectors in the absence of the polyC-tract; therefore, evidence that the polyC tract directly recruits factors indispensable for translation is currently lacking. However, comparative studies conducted in both cell-free systems and cell lines demonstrated that in EMCV- and FMDV-derived vectors, type II IRES–mediated translation efficiency is heavily affected by reaction parameters as well as the species and tissue of origin of the cell line used [82,83]. Furthermore, deviations from the wild-type sequence of the EMCV IRES, even when minimal, can also cause suboptimal translation efficiency [84]. Comparative studies of type II IRES–mediated translation in EMCV, Mengovirus, or FDMV in tissues and cell types where polyC-mediated restrictions have been reported would be necessary to fully understand the possible contribution of the polyC tract to translation. Experimental design can be guided by existing interaction data. Since the polyC tract is not an absolute requirement for viral viability, these potential RNA–protein interactions most likely play a cell type–restricted stimulatory/enhancing role. Many cellular factors recruited on viral RNA genomes, particularly those involved in replication and translation, are proteins involved in mRNA maturation, stabilization, splicing, and transport that get redirected from their nuclear localization to the cytoplasm upon infection [85]. Intriguingly, the 2 most well-known proteins recruited on picornaviruses genomes recognize and bind polyU/C-rich sites. The better studied—and first to be identified—of these proteins is the polypyrimidine tract–binding protein (PTB), which participates in the alternative processing and translation of various cellular RNAs and binds short sequences rich in uridine and cytosine residues, UCUU(C) and UUCU/C [86]. During picornavirus infection, PTB is a required IRES trans-activating factor (ITAF) that guides the assembly of the translational machinery on the 5′-NCR of EMCV, FMDV, TMEV, poliovirus, and Coxsackievirus B3 [87–90]. A second family of interesting proteins is the poly(rC)-binding protein (PCBP), members of which are involved in the stabilization of several cellular mRNAs and bind nucleic acids through hnRNP k-homologous domains [91]. A member of this family in particular, PCBP2, has been extensively described in association with picornaviruses because it plays fundamental roles in both translation and replication. As an ITAF essential for translation, PCBP2 directly binds to C-rich bulges of arms *a* and *b* of the stem-loop IV in type I IRESs in poliovirus, Coxsackievirus B3, and rhinovirus [92–95]. PCBP2 interacts with type II IRESs on EMCV and FMDV, but it is not strictly required for translation [95]. Even though it is not known whether PTB or PCBP2 can interact directly with the polyC tract, it is certainly interesting to speculate about the possibility of such proteins using the polyC tract as a landing pad to enhance translation. Cell type–specific expression of ITAFs has also been shown to regulate and restrict viral replication in certain tissues, which would explain the cell and tissue type–specific relevance of the polyC tract for replication observed in Mengovirus, EMCV, and FMDV [90,96].

Even more interestingly, PCBP2 also binds to C-rich motifs on bulge *b* in the cloverleaf (ACCCA) and in a spacer sequence immediately downstream of the cloverleaf (U/ACCC(CC)UCCCCCA) of *Enteroviruses* [92,97,98]. This interaction is fundamental for the synthesis of both the negative- and positive-sense genomes, as it allows the circularization of the template RNA: Viral protein 3CD binds to bulge *d* of the cloverleaf and to PCBP2, forming a ternary complex that bridges the gap with the polyA-binding protein (PABP) associated with the polyA on the 3′-terminal [9,99]. The circularization is a fundamental step to allow the switch from translation to replication and provide the viral RNA-dependent RNA polymerase access to the VPg primer. *Cardioviruses* and *Aphthoviruses* do not possess a cloverleaf structure, which poses the question of how do these viruses accomplish circularization. In FMDV, PCBP2 promotes viral replication [100]. Moreover, FMDV S-fragment has been shown to bind 3CD and PCBP1/2 [101]. With these premises, it is interesting to imagine the S-fragment of *Cardioviruses* and *Aphthoviruses* playing the same role the cloverleaf plays in *Enteroviruses* by recruiting the circularization complex at the 5′-terminus, with the polyC tract working as C-rich spacer region immediately downstream for the stabilization of PCBP2 binding. Further validation of this hypothesis comes from the fact that mutations in the spacer region of poliovirus also abolish neurovirulence and attenuate the virus [102].

Another hypothesis is that the PolyC tract could have evolved to counteract the type I IFN antiviral response. PolyU/UC sequence signatures on the *Flavivirus* hepatitis C virus (HCV) NCRs have been shown to be specifically recognized by cytosolic RNA sensor RIG-I [103]. Even though it might seem counterintuitive, a motif capable of binding innate immune sensors could work in favor of the virus by acting as a molecular sink or decoy for MDA-5 and protein kinase R (PKR), both cytosolic RNA sensors known to recognize *Cardioviruses* and *Aphthoviruses* [41,74,104]. Recent studies identified structural motifs in *Alphavirus* 5′-NCRs capable of avoiding immune restriction by antiviral factor IFIT1 through alterations of its binding and function, even though the molecular mechanism has not been elucidated yet [105]. Given the relevance of the polyC tract for replication in immune cells, a role in escaping the antiviral response can be imagined.

The function of the polyC tract might be not to act as a binding motif for protein factors but to act as a structural element that allows the RNA genome to assume more favorable spatial conformations. Although additional structural studies are needed, polyC tracts are not currently known to fold into any secondary structures and are thought to loop out of an otherwise highly organized 5′-NCR [39,40]. This long and flexible stretch of the genome could simply be a linker region between other rigid RNA structures. It is not unfounded that alteration of polyC length could impact RNA–RNA or RNA–protein interactions of the surrounding structural elements in a cell-specific manner. Alternatively, the polyC tract might help the spatial organization of the genome by interacting with other RNA structures or motifs within the viral genome. Such long-range, protein-independent RNA interactions have been reported for FMDV between the 2 stem-loops of the 3′-NCR and the 2 main structures of the 5′-NCR, the IRES and the S-fragment [101]. Since the interaction between the stem-loops and the S-fragment is stronger than the one between the stem-loops and the IRES, the authors propose that such interactions could be responsible for the switch between 3′-NCR/IRES–driven translation to 3′-NCR/S-fragment–driven replication [101]. This scenario provides an alternative solution to the problem of circularization that both *Cardioviruses* and *Aphthoviruses* face, in which the polyC could provide further stabilization to the 3′-NCR/S-fragment interaction.

Finally, it is also possible that the polyC sequence per se is not the molecular motif triggering the mechanism of attenuation: The complementary polyguanosine (polyG) sequence present at the 3′-NCR of the negative-strand or even the double-stranded RNA stretches of polyC/G that come into existence temporarily during the replicative form (RF) and the replicative intermediate (RI) might also be the culprit. The use of cytidines is clearly important because replacing it with polyU or random filler has the same effect of a total deletion [59]. To this point, it is interesting to consider that any double-stranded RNA sequence so rich in C/G pairing—if allowed to form—would be extremely hard to pull apart at every cycle of replication but would also steady the replication forms against any attempt from cell factors to interfere with genome synthesis. Double-stranded RNAs rich in G/C and longer than 30 nucleotides are also very good activators of PKR and MDA-5 [106–108].

## Applications

PolyC truncations to control the virulence of EMCV-Mengovirus and FMDV have obvious practical applications for the containment of the viruses themselves, which are known pathogens for swine, cloven-hoofed, and zoological animals [49,109]. Vaccination studies using MC_0_ and MC_24_ have repeatedly shown that inoculation with short-tract Mengoviruses is protective against infection with wild-type Mengovirus and EMCV [69,70]. These live-attenuated Mengovirus variants even perform better in a direct comparison with the available EMCV inactivated vaccine, which requires repeated administrations with an adjuvant to induce neutralizing antibodies and provide protection [69]. This striking immunogenicity of short-tract Mengovirus also extends to heterologous antigens cloned within the L protein sequence, which are capable of inducing a protective cytotoxic T cell–mediated immune response against the epitope [110]. However, the potential of short-tract Mengovirus as vaccine platform for other pathogens is greatly limited by the size of the antigens that can be inserted in Mengovirus genome without affecting its stability [111]. Because of truncated Mengovirus impressive performance as vaccine, polyC function was also investigated in FMDV as a possible strategy for the development of a live vaccine alternative. However, early evidence of the genetic instability of short polyC tract in the FMDV genome quickly hampered the enthusiasm for the possibility of a polyC-truncated or deleted live FMDV vaccine.

Modulating viral pathogenicity in replication-competent viruses is also an important requirement for OV, a fairly novel platform for cancer therapy. This approach harnesses the innate propensity of viruses to selectively infect and lyse cancer cells for therapeutic purposes [112]. Because of their small size, rapid cytosolic replication cycle, and high yield and easy-to-manipulate genome, several picornaviruses are under investigation for their potential as oncolytic viruses [113]. The 2 most prominent candidates for the use of picornavirus in cancer therapy are 2 *Enteroviruses*, Coxsackievirus A21 (CAV21, registered as CAVATAK) and a chimeric poliovirus-human rhinovirus (PVS-RIPO), both currently in Phase I and II clinical trials as therapeutics for a variety of tumor types, alone, or in combination with immunomodulators (http://clinicaltrials.gov). One of the most important conundra in OV revolves around the necessity to balance safety and efficacy: Efforts to reduce pathogenicity in order to control replication and limit side effects for the patients and transmission risks for the population might end up compromising potency and immunogenicity [114]. Notably, the use of the human pathogen poliovirus as an oncolytic virus under the PVS-RIPO formulation is only possible because modulation of RNA secondary structures in the 5′-NCR allows tissue-specific attenuation. PVS-RIPO is in fact a chimeric virus consisting of poliovirus Sabin 1 strain backbone and the IRES of human rhinovirus 2 (HRV2), which is recognized by the double-stranded RNA binding protein 76 (DRBP76) dimerized with nuclear factor of activated T cells 45 kDa (NF45). DRBP76-NF45 heterodimers present in neuronal cells but not in glioma cells recognize and bind the IRES of PVS-RIPO, inhibiting translation in healthy tissues while allowing replication in tumor cells [96,115]. Because of their positive-sense RNA genomes, oncolytic picornaviruses are especially suitable for another form of post-viral entry replication control, which hijacks the miRNA gene expression control network to regulate viral permissivity. miRNA-targeting elements inserted within the viral genome target picornaviruses for degradation in healthy tissues expressing the cognate miRNA, while replication occurs unencumbered in neoplastic tissues where the cognate miRNA is not expressed [116]. This approach was shown to reliably eliminate fatal CAV21-associated myotoxicity in OV mouse models, both in the infectious RNA and viral particle formulation [117–119]. The use of miRNA-targeting elements not only controls oncolytic picornavirus permissivity and hence tissue-specific toxicities, but also provides an excellent safety platform to investigate the impact on oncolytic activity of other genome elements. This opportunity comes especially in handy with Mengovirus. Short-tract Mengovirus MC_24_ has proven to be able to effectively infect and debulk tumor volume in a multiple myeloma model, but the neuronal and cardiac toxicity associated with high titers needed for oncolytic therapy were detrimental to mice survival [51]. miRNA-targeting elements recognized by miRNA overexpressed in the brain and cardiac muscular tissues inserted in MC_24_ NCRs (MC_24_-NC) significantly reduced replication in the target tissues even at extremely high titers and increased the safety profile of MC_24_. Oncolytic therapy with MC_24_-NC was able to delay tumor growth and even completely clear tumors in multiple myeloma models, but its efficacy is not consistent across the board [51,120]. Through the use of miRNA-targeting platform, it is now possible to reintroduce the full-length polyC tract in Mengovirus and investigate the oncolytic potential of long-tract Mengovirus while preserving safety. In this context, understanding the mechanism of action of the polyC tract and its association with pathogenicity in Mengovirus has the twofold advantage of increasing the arsenal for clinical translation of OV and shed some light on the correlation between efficacy and virulence.

## Concluding remarks

The polyC tract is a unique element of picornavirus biology whose molecular function and connection to pathogenesis has eluded explanation so far. The great variability in terms of genetic stability, impact on viral viability, and virulence hints at a mechanism that is far more complex than the simplicity of this RNA element molecular design—just a stretch of cytidine residues, might lead to believe. The idea that a few nucleotides difference in length is all that these viruses need to make a difference between life and death is deeply fascinating per se, but also has deep implication on the way we think about RNA viruses. Picornaviruses, just like all small RNA viruses, cannot rely on a plethora of protein-encoding genes to perform all the functions needed to hijack and defeat host cell defenses and have therefore evolved to maximize function and information embedded in their genomes to the point that they can achieve lethality by just increasing a polyC tract by a dozen nucleotides. Investigating the RNA elements distributed along the whole length of picornavirus genomes can increase our knowledge of what kind of functions are possible for RNA molecules in general. This kind of analysis has brought the world of molecular biology numerous tools that are now extensively used to parse out biological processes—a classic example being another fundamental picornavirus structure, the IRES, now extensively used to generate polycistronic reporter genes assays. Contrary to IRES, the polyC tract is unique to few picornavirus species belonging to diverse genera, which brings the question as to why this RNA element evolved specifically in the genomic context of *Aphthovirus* and *Cardiovirus*. The mystery of the polyC tract is actually 2-fold: what are the molecular interactions involving the polyC tract happening intracellularly that impact viral replication and how does the outcome of such molecular interactions ripple down to pathogenicity. Research on this topic has mostly been held back on one hand by the intrinsic difficulty of accurately sequencing long stretches of repetitive sequence and on the other hand by the challenge of synthesizing, cloning, and maintaining long CG tracts in bacterial plasmids. These obstacles explain why it has not been easy to investigate the polyC, especially at the time of its first description almost 50 years ago, when the best tool to assess the length of the polyC was digestion with RNases. We are now in the position to shed light on this somewhat forgotten enigma, a task that has become increasingly important. Studying virulence factors in animal pathogens has an immediate application for prevention and vaccination efforts, especially for economically relevant viruses such as FMDV and EMCV, but the benefits are not limited to animal welfare. Broadening the knowledge on pathogenicity determinants in other mammals has a huge but less direct impact on human health too: first, because it leaves us better equipped to understand and deal with the threats of zoonosis and emerging pathogens, and, second, because it gives us the tools to fine-tune the design of viral vectors for applications such as OV, where the balance between safety and efficacy is based on harnessing and giving purpose to viruses’ natural inclination for destruction.
